# Supplementary material for: Auditory deep sleep stimulation in older adults at home: a randomized crossover trial
Source: Commun Med (Lond). 2022 Apr 4;2:30. doi: 10.1038/s43856-022-00096-6 (PMC9053232; doi:10.1038/s43856-022-00096-6)
Supplement: Supplementary file 1 — Reporting Summary [file 43856_2022_96_MOESM1_ESM.pdf]

nature research

Corresponding author(s): Caroline Lustenberger

Last updated by author(s): Mar 3, 2022

Reporting Summary

Nature Research wishes to improve the reproducibility of the work that we publish. This form provides structure for consistency and transparency in reporting. For further information on Nature Research policies, see our Editorial Policies and the Editorial Policy Checklist.

Statistics

For all statistical analyses, confirm that the following items are present in the figure legend, table legend, main text, or Methods section.

n/a	Confirmed	
☐	☒	The exact sample size (*n*) for each experimental group/condition, given as a discrete number and unit of measurement
☐	☒	A statement on whether measurements were taken from distinct samples or whether the same sample was measured repeatedly
☐	☒	The statistical test(s) used AND whether they are one- or two-sided<br>*Only common tests should be described solely by name; describe more complex techniques in the Methods section.*
☒	☐	A description of all covariates tested
☐	☒	A description of any assumptions or corrections, such as tests of normality and adjustment for multiple comparisons
☐	☒	A full description of the statistical parameters including central tendency (e.g. means) or other basic estimates (e.g. regression coefficient) AND variation (e.g. standard deviation) or associated estimates of uncertainty (e.g. confidence intervals)
☐	☒	For null hypothesis testing, the test statistic (e.g. *F*, *t*, *r*) with confidence intervals, effect sizes, degrees of freedom and *P* value noted<br>*Give P values as exact values whenever suitable.*
☒	☐	For Bayesian analysis, information on the choice of priors and Markov chain Monte Carlo settings
☐	☒	For hierarchical and complex designs, identification of the appropriate level for tests and full reporting of outcomes
☐	☒	Estimates of effect sizes (e.g. Cohen's *d*, Pearson's *r*), indicating how they were calculated

Our web collection on statistics for biologists contains articles on many of the points above.

Software and code

Policy information about availability of computer code

Data collection	EEG/stimulation parameters were recorded using the MHSL-Sleepband v2.0 (Ferster et al. 2019, IEEE Sensors letter). Android Apps build with lambdanative were used to collect phone data. Data collected by phone was directly transferred and stored in REDCap. Other questionnaire assessments were collected directly via REDCap surveys on a browser.
Data analysis	All data was analyzed using Matlab and the open source "R" software package. In Matlab, standard functions such as pwelch were used to extract EEG power values (e.g. lowSWA), preprocessing of EEG data (filtering) was performed with EEGLAB toolbox. Machine learning models for automatic sleep stage scoring were built using Matlab, Python and Tensorflow. Statistics were performed using R and available libraries including robustlmm, prkbtest, ggplot2, rmcorr, ordinal, caret

For manuscripts utilizing custom algorithms or software that are central to the research but not yet described in published literature, software must be made available to editors and reviewers. We strongly encourage code deposition in a community repository (e.g. GitHub). See the Nature Research guidelines for submitting code & software for further information.

Data

Policy information about availability of data

All manuscripts must include a data availability statement. This statement should provide the following information, where applicable:

- Accession codes, unique identifiers, or web links for publicly available datasets
- A list of figures that have associated raw data
- A description of any restrictions on data availability

Source data for the main figures in the manuscript can be accessed as Supplementary Data 1-4. Raw data that support the findings of this study cannot be made publicly available to protect participants' rights according to Swiss human research law. The de-identified individual participant data that underly the results of this paper can be accessed by investigators who (1) submit a methodological sound proposal describing the intended analysis and as reviewed by the authors of this

publication, (2) provide proof of relevant ethical approval for the intended analysis, and (3) fulfill data protection measures according to Swiss legal requirements. The analyses of the shared data is restricted to achieve the aims of the intended analysis. Along with the data, no other documents will be made available. Proposals with reasonable requests may be submitted to walter.karlen@ieee.org up to 24 months following article publication.

Field-specific reporting

Please select the one below that is the best fit for your research. If you are not sure, read the appropriate sections before making your selection.

☒ Life sciences ☐ Behavioural & social sciences ☐ Ecological, evolutionary & environmental sciences

For a reference copy of the document with all sections, see nature.com/documents/nr-reporting-summary-flat.pdf

Life sciences study design

All studies must disclose on these points even when the disclosure is negative.

Sample size	For the performed interim analysis that is reported here, our sample size was based on previously published lab studies that showed in a within subject design, a significant effect of auditory stimulation on slow waves. Several landmark papers had ~13 participants that reported a significant effect on sleep upon auditory stimulation in different age groups (e.g. Young: Ngo et al, 2013 Neuron; Besedovsky et al., 2017, Nature Communications; Elderly: Papalambros et al. 2017, Front. Hum. Neurosci.). We used 16 participants to match with this number and to allow for a counter-balanced study design.
Data exclusions	Overall, 33 participants were enrolled and screened. Here, we report data of 16 participants as part of an interim analysis that underwent the whole experimental procedure of auditory up-phase slow wave stimulation over multiple weeks and had sufficient data for analysis. Of the 33 enrolled participants, 17 received allocated up-phase intervention and sham in a counter-balanced design, however one female participant (sham on first, not mentioned in randomization tab below) had 2 weeks of completely missing sleep data due to wrong use of device. The remaining participants did either not pass the screening phase (exclusion criteria, withdrawn), did not receive allocated up-phase intervention, or did withdraw after a few nights of the experiments (see Supplementary Figure 1 in manuscript for details). In the included 16 participants some nights/tasks/questionnaires had to be excluded due to bad data quality or missing data. An overview of included assessments (number) is summarized in Supplementary Table S3 in the paper.
Replication	Our multiple nights of stimulation, and the two stimulation protocols that target the up-phase of sleep slow waves (just dose-dependent differences) provide robust findings because (1) across multiple nights the effect on SWA was seen and (2) findings in windowed stimulation approach are reproduced in continuous approach
Randomization	Randomization was performed by the study coordinator that was not involved in recruitment or data collection but only analysis. Participants were randomized in terms of which intervention condition will take place first, having 4 different options (Verum ON, Verum ONOFF, Sham ON, Sham ONOFF) using a blocked design to guarantee balance. After the first condition was defined, the following conditions were set (sham always followed sham, and ON/ONOFF were kept in the same order, the same was for verum). Gender was taken into account when performing randomization in order to achieve an equal gender balance. The randomization order was predefined in blocks for both genders before study start and then participants were randomly allocated by the study coordinator to the first condition in sequence after their successful enrollment upon passing screening performed by trained and blinded research assistants. The randomization aimed at 25% (n=4) start per condition, however due to intermediate drop-outs (see data exclusion above) and gender imbalance, this counter-balance was not perfectly achieved but very close to the aimed distribution: Start Sham ONOFF: 4 (2f, 2m), Sham ON: 3 (1f, 2m), Verum ONOFF: 5 (2f, 3m), Verum ON: 4 (2f, 2f).
Blinding	The participants and experimenters performing the assessments and providing the device/instructions were blinded to the conditions. Study coordinator, PI and lead engineer had to know condition for device settings and safety reasons. Experimenters performing the assessments were blinded to the performed intervention by restricting access to device settings only to the lead engineer and randomization list access only to the study coordinator. Participants were blinded by having for both sham and verum conditions exactly the same application procedures of the device and the exact same physical appearance of the device independent of intervention setting. Analysis of sleep stages (manual re-scoring of a few automatically labeled sleep epochs) was done by an expert not involved in the study, and he was not informed about conditions and intention of the study.

Reporting for specific materials, systems and methods

We require information from authors about some types of materials, experimental systems and methods used in many studies. Here, indicate whether each material, system or method listed is relevant to your study. If you are not sure if a list item applies to your research, read the appropriate section before selecting a response.

Materials & experimental systems

n/a	Involved in the study	
☒	☐	Antibodies
☒	☐	Eukaryotic cell lines
☒	☐	Palaeontology and archaeology
☒	☐	Animals and other organisms
☐	☒	Human research participants
☒	☐	Clinical data
☒	☐	Dual use research of concern

Methods

n/a	Involved in the study	
☒	☐	ChIP-seq
☒	☐	Flow cytometry
☒	☐	MRI-based neuroimaging

Human research participants

Policy information about studies involving human research participants

Population characteristics	Participants between the age of 60-84 were allowed to participate. All participants were in good general health, non-smokers. None of them had a presence or history of a psychiatric/neurologic disorder, presence of a diagnosed sleep disorder or internal disorder. Participants were further excluded if they used on-label sleep medication.
Recruitment	The participants were recruited from the community using advertisements on different platforms/newspapers and at meet-ups. The recruitment was further supported by the University Zurich Research Priority Program "Dynamics of Healthy Aging" and the Senior University.
Ethics oversight	The study was approved by the cantonal Ethics Committee Zurich (reference number: KEK ZH, BASEC 2017-01436), the Swiss Agency for Therapeutic Products Swissmedic (reference number: 10000181/ 2017-MD-0027) and was conducted in accordance with the Declaration of Helsinki. The study was registered at ClinicalTrials.gov (NCT03420677).

Note that full information on the approval of the study protocol must also be provided in the manuscript.
