# Supplementary material for: Auditory deep sleep stimulation in older adults at home: a randomized crossover trial
Source: Commun Med (Lond). 2022 Apr 4;2:30. doi: 10.1038/s43856-022-00096-6 (PMC9053232; doi:10.1038/s43856-022-00096-6)
Supplement: Supplementary file 2 — Description of Additional Supplementary Files [file 43856_2022_96_MOESM2_ESM.pdf]

Description of Additional Supplementary Files

File Name: Supplementary Data 1

Description: Source data for main Figure 2 in the manuscript.

File Name: Supplementary Data 2

Description: Source data for main Figure 3 in the manuscript.

File Name: Supplementary Data 3

Description: Source data for main Figure 4 in the manuscript.

File Name: Supplementary Data 4

Description: Source data for main Figure 5 in the manuscript.
