# Supplementary material for: Auditory deep sleep stimulation in older adults at home: a randomized crossover trial
Source: Commun Med (Lond). 2022 Apr 4;2:30. doi: 10.1038/s43856-022-00096-6 (PMC9053232; doi:10.1038/s43856-022-00096-6)
Supplement: Supplementary file 3 — Supplementary Information [file 43856_2022_96_MOESM3_ESM.pdf]

Supplementary Information: Figures and Tables

Auditory deep sleep stimulation in older adults at home: a randomized crossover trial

Caroline Lustenberger[1,2,3*], M. Laura Ferster[4], Stephanie Huwiler[1], Luzius Brogli[4,5], Esther Werth[2,3,6], Reto Huber[2,3,7,8], Walter Karlen[3,4,5]

[1] Neural Control of Movement Lab, Institute of Human Movement Sciences and Sport, Department of Health Sciences and Technology, ETH Zurich, Zurich, Switzerland

[2] Neuroscience Center Zurich (ZNZ), University of Zurich and ETH Zurich, Zurich, Switzerland

[3] Center of Competence Sleep & Health Zurich, University of Zurich, Zurich, Switzerland

[4] Mobile Health Systems Lab, Institute of Robotics and Intelligent Systems, Department of Health Sciences and Technology, ETH Zurich, Zurich, Switzerland

[5] Institute of Biomedical Engineering, Universität Ulm, Ulm, Germany

[6] Department of Neurology, University Hospital Zurich, University of Zurich, Zurich, Switzerland

[7] Child Development Centre, University Children's Hospital, University of Zurich, Zurich, Switzerland

[8] Department of Child and Adolescent Psychiatry and Psychotherapy, Psychiatric Hospital Zurich, University of Zurich, Zurich, Switzerland

*Present address of corresponding Author: Caroline Lustenberger, Neural Control of Movement Lab, Department of Health Sciences and Technology, ETH Zurich, Zurich, 8092, Switzerland. Email: caroline.lustenberger@hest.ethz.ch

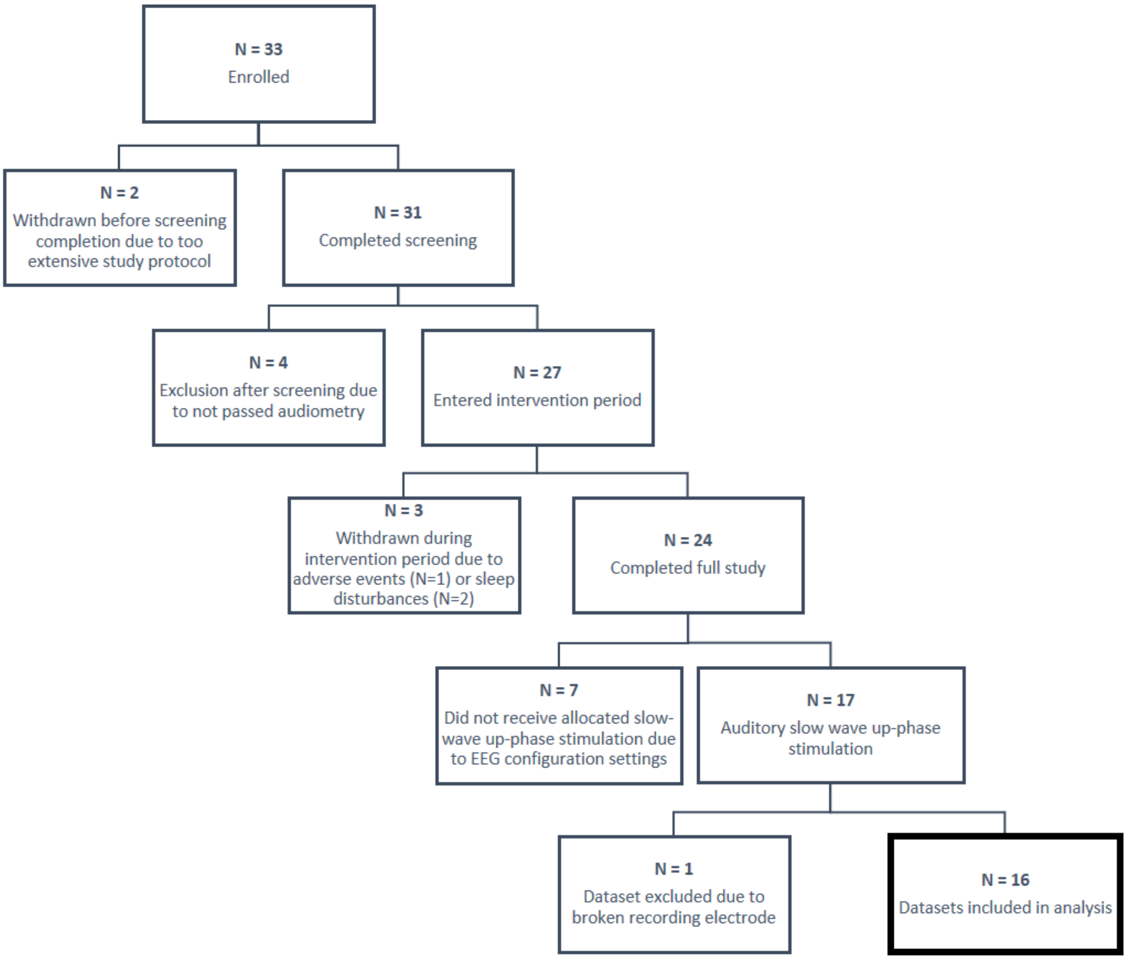

Figure S1: Trial profile for the study. Overview of enrolled participants (after signed consent form).

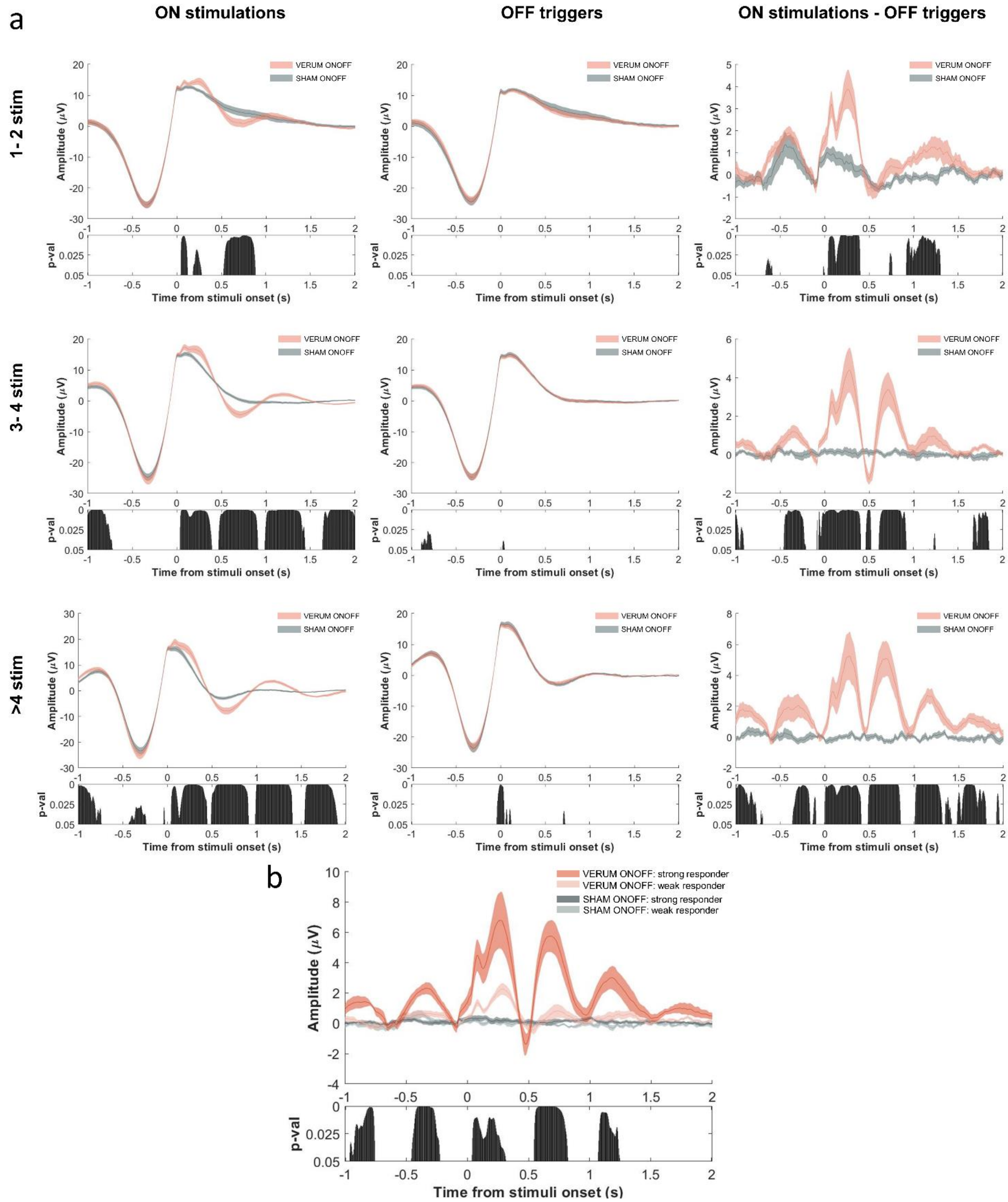

Figure S2: Evoked responses (ERP) for verum and sham nights for the windowed approach with statistical results of linear mixed-effect models. (a) ERPs between sham and verum nights separately for ON (1st column), OFF (2nd column) and ON-OFF difference (3rd column), all for different stimulation numbers (row 1-3). P-values refer to the outputs of linear mixed-effect models for the factor condition (verum vs. sham). (b) ERP for all stimuli (tones or triggers) across conditions separately for weak and strong responders. P-values from linear mixed-effect model refer to the interaction condition x responder.

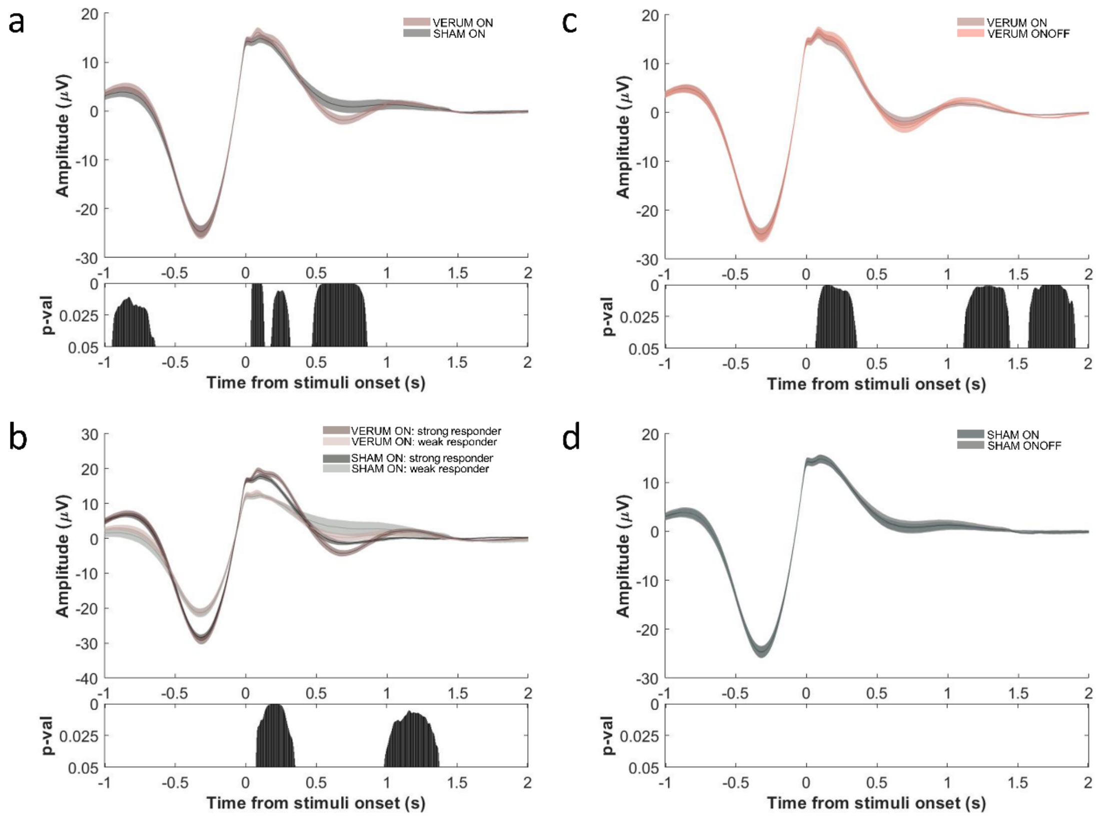

Figure S3: Evoked responses (ERP) for verum and sham nights with statistical results of linear mixed-effect models. (a) ERPs between sham and verum nights for the continuous approach including all stimulations (verum) or triggers (sham). P-values refer to the outputs of linear mixed effects models for the factor condition (verum vs. sham). (b) ERP for all stimuli (tones or triggers) across conditions separately for weak and strong responders for the continuous approach. P-values from linear mixed-effect model refer to the interaction condition x responder. (c) and (d) illustrate ERPs for all stimulations between the windowed and continuous approach for verum (c) and sham (d). P-values from linear mixed-effect model refer to the factor approach (ON vs ONOFF).

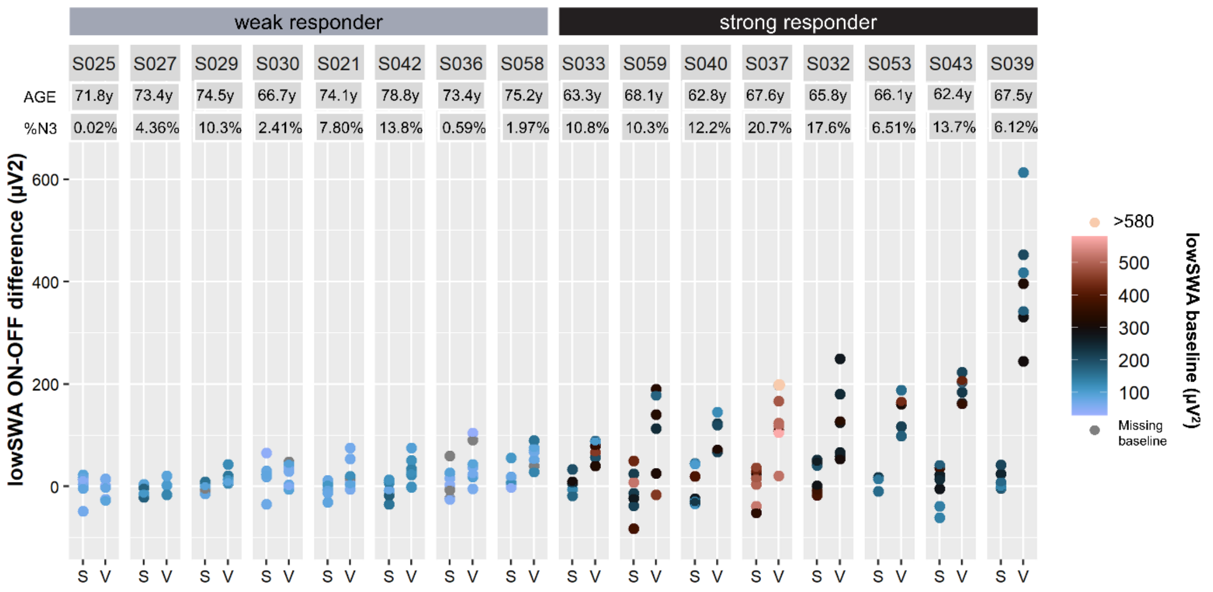

Figure S4: Individual low-frequency slow wave activity (SWA) response to auditory stimulation in relation to baselineSWA. Individual ($n_{Subjects}$ = 16) ON-OFF difference of lowSWA for all windows with >2 stimulations sorted by averaged lowSWA during verum in the windowed approach. Strong responders refer to the upper 50% and weak responder to the lower 50% of participants. Nights are color coded by the amount of lowSWA baseline activity. Baseline activity corresponds to the first 10 min of stable non-rapid eye movement (NREM) sleep detected during which the device was not performing stimulations. Age and averaged percentage of NREM sleep stage N3 (%N3) per night are summarized below each participant label. S: sham V: verum.

Table S1: Performance metrics of auditory stimulation algorithm

	Windowed (ONOFF) approach		Continous (ON) approach		All conditions
	Sham	Verum	Sham	Verum	
**Performance metric**	*Mean (SEM)*	*Mean (SEM)*	*Mean (SEM)*	*Mean (SEM)*	*Mean (SEM)*
Sleep classification					
Precision	0.87 (0.02)	0.87 (0.02)	0.86 (0.02)	0.88 (0.02)	0.87 (0.02)
Specificity	0.87 (0.02)	0.87 (0.02)	0.87 (0.02)	0.87 (0.02)	0.88 (0.02)
Recall	0.51 (0.05)	0.53 (0.05)	0.52 (0.05)	0.55 (0.05)	0.52 (0.05)
Stimulation triggers					
Phase mean (°)	42.6 (1.03)	40.6 (0.92)	41.74 (1.50)	41.46 (0.89)	41.7 (0.85)
Phase standard deviation (°)	44 (0.66)	42.1 (0.73)	44.5 (0.93)	43 (0.99)	43.3 (0.71)
Phase median (°)	39.6 (0.98)	39.2 (1.25)	39 (1.08)	39.3 (1.1)	39.3 (0.85)
NREM sleep (#)	1734 (227)	1849 (262)	3488 (544)	3764 (545)	2645 (377)
not-NREM sleep (#)	62 (17)	59 (15)	117 (32)	138 (46)	90 (26)
Precision w.r.t. NREM sleep	0.96 (0.01)	0.96 (0.01)	0.96 (0.01)	0.96 (0.01)	0.96 (0.01)
Volume					
Mean (dB)	55 (0.53)[a]	54.7 (0.54)	55 (0.61)[a]	55 (0.65)	54.93 (0.58)[b]
Minimum (dB)	51.9 (0.03)[a]	51.9 (0.03)	52 (0.02)[a]	51.9 (0.07)	51.9 (0.03)[b]
Maximum (dB)	58.2 (0.78)[a]	57.9 (0.82)	58.6 (0.65)[a]	58.3 (0.72)	58.2 (0.74)[b]

a: hypothetical volume (0 dB was applied during sham), b: only includes verum nights. Precision, specificity, and recall are provided as relative values ranging from 0 to 1.

Table S2: Summary of included assessments per approach and condition

	Windowed (ONOFF) approach		Continous (ON) approach	
	**Sham**	**Verum**	**Sham**	**Verum**
**Assessment**	*# nights (# subject)*	*# nights (# subject)*	*# nights (# subject)*	*# nights (# subject)*
Sleep stages	98 (16)	102 (16)	92 (15)	94 (15)
Spectral analysis	98 (16)	102 (16)	92 (15)	97 (15)
Sleep quality assessments	97 (16)	102 (16)	92 (15)	97 (15)
Mood assessments morning	97 (16)	102 (16)	92 (15)	97 (15)
Mood assessment 8pm	79 (16)	80 (16)	72 (15)	74 (15)
KSS morning	97 (16)	102 (16)	92 (15)	97 (15)
KSS 2pm	84 (15)	82 (16)	73 (15)	83 (15)
KSS 8pm	79 (16)	80 (16)	72 (15)	74 (15)
PVT 2pm	82 (15)	79 (15)	72 (15)	77 (15)

KSS: Karolinska Sleepiness Scale, PVT: Psychomotor vigilance test.

Table S3: **Sleep architecture and arousal for windowed and continuous approach**. Robust linear mixed models were performed to compare sham to verum nights. P-value of factor Condition (sham vs verum) and the interaction of condition (cond) and responder (resp, weak vs. strong) for each approach is reported in the statistics columns. Significant and trend-level p-values are marked with an asterisk (*).

	Windowed (ONOFF) approach				Continous (ON) approach			
	Sham	Verum	statistics		Sham	Verum	statistics	
	Mean (SEM)	Mean (SEM)	p_{cond}	$p_{condxresp}$	Mean (SEM)	Mean (SEM)	p_{cond}	$p_{condxresp}$
Sleep architecture								
Total time in bed (min)	498.8 (13.7)	493.3 (13.5)	0.50	0.19	501.3 (12.5)	502.4 (14.7)	0.98	0.24
Sleep efficiency (%)	81 (1.7)	81.4 (1.8)	0.52	0.41	83.2 (1.8)	80.9 (2.2)	0.18	0.15
Sleep latency (min)	27.4 (4.7)	21 (2.4)	0.043*	0.047*	27.7 (5.0)	25.2 (3.5)	0.31	0.013
NREM N1 (min)	48.4 (4.0)	46.9 (5.4)	0.65	0.39	49.0 (5.0)	50.7 (4.6)	0.79	0.65
NREM N2 (min)	217.3 (11.8)	219.8 (9.9)	0.90	0.42	226.0 (11.4)	220.1 (12.7)	0.28	0.40
NREM N3 (min)	41.3 (7.7)	45.4 (7.8)	0.17	0.082*	41.2 (7.7)	43.3 (9.3)	0.35	0.11
REM latency (min)	98.9 (8.5)	93.5 (5.9)	0.36	0.068*	102.7 (9.2)	104.1 (8.4)	0.97	0.63
REM (min)	98.5 (5.8)	91.8 (5.6)	0.059*	0.91	101 (4.7)	94.1 (7.1)	0.075*	0.083*
WASO (min)	67.4 (6.4)	69.5 (6.6)	0.62	0.54	58.3 (7.1)	71.1 (8.4)	0.040*	0.021*
Arousal								
NREM N1-N3 (#)	49.3 (6.6)	52.7 (7.6)	0.19	0.77	45.4 (6.1)	45.5 (7.1)	0.76	0.15
REM (#)	13.6 (2.7)	13.1 (2.3)	0.77	0.44	13.5 (2.6)	13.9 (2.7)	0.82	0.96
